# Imaging Through Scattering Tissue Based on NIR Multispectral Image Fusion Technique

**DOI:** 10.3390/s25164977

**Published:** 2025-08-12

**Authors:** Nisan Atiya, Amir Shemer, Ariel Schwarz, Yevgeny Beiderman, Yossef Danan

**Affiliations:** 1 Department of Electrical and Electronics Engineering, Azrieli College of Engineering, Jerusalem 9103501, Israel; nisanat@post.jce.ac.il (N.A.); arielsc@jce.ac.il (A.S.); yossefda@jce.ac.il (Y.D.); 2 Faculty of Electrical and Electronics Engineering, Holon Institute of Technology, Holon 5810201, Israel; beidermany@hit.ac.il

**Keywords:** multispectral imaging, image fusion, scattering tissue imaging, pyramid decomposition

## Abstract

Non-invasive diagnostics play a crucial role in medicine, and they ensure both contamination safety and patient comfort. The proposed study integrates hyperspectral imaging with advanced image fusion, enabling non-invasive, diagnostic procedure within tissue. It utilizes near-infrared (NIR) wavelength vision that is suitable for reflections from objects within a dispersive layer, enabling the reconstruction of internal tissue layers images. It can detect objects, including cancerous tumors (presented as phantoms), inside human tissue. This involves processing data from multiple images taken in different NIR bands and merging them through image fusion techniques. Our research demonstrates evident data about objects within the diffusive media, visible only in the reconstructed images. The experimental results demonstrate a significant correlation with the samples employed in the study’s experimental design.

## 1. Introduction

The identification of clear tumor margins is a critical factor in the complete removal of tumors and reducing the risk of their recurrence [1]. This is particularly important when the tumor is located near neurological structures, as removing too much tissue can be dangerous and harmful to vital organs or nerve tissue [2]. Several methods are aimed at improving tumor margin visualization, including MRI, CT, and targeted fluorescence imaging [1,3,4,5,6,7,8,9]. However, these methods have limitations such as reduced spatial resolution due to tissue shifting during surgery in MRI and CT, dependence on photo-physics and photochemistry in targeted fluorescence imaging, as well as issues such as phototoxicity and photobleaching [10,11,12,13,14,15,16]. Moreover, MRI and CT devices are costly and less accessible to common patients. The CT technique can also be harmful to the human body due to its radiative detection nature, and a major concern with this medical imaging is the risk from ionizing radiation, such as X-rays and gamma rays, which can increase the likelihood of DNA damage and cancer.

Utilizing a hyperspectral camera within the near-infrared spectrum introduces a novel approach aimed at identifying reflections originating from objects situated within a dispersive layer. By processing data from images captured in various NIR bands and utilizing image fusion techniques [17], one can produce a non-invasive and non-harmful tumor detection method. This technique holds promise for detecting tumors located inside human tissue layers.

The human body’s tissues, rich in fluids, exhibit dispersion, contrasting with the dense composition of tumor tissue. When directing halogen light sources with a black-body radiation spectrum toward the body, the heightened density of tumors yields significantly more reflections compared to a typical healthy tissue [18,19]. Conventional silicon-based cameras primarily function within the visible spectrum range of 400–700 [nm] and are much less sensitive to NIR spectrum [20]. Yet, detecting objects obscured by diffuse tissue in this spectrum proves challenging. This limitation arises from the scattering or absorption of much of the visible light within the tissue, preventing deep penetration. A hyperspectral camera in the NIR range can be used to sense light coming from tissue at a depth of up to 2 cm; thus, cancerous tumors and blood vessels can be detected [21,22].

In the proposed work, the illumination for the hyperspectral imaging is achieved using a halogen light, which is an incandescent lamp (that generates light by heating a solid body to a high temperature) [23]. Halogen illumination was chosen because it has wide-spectrum illumination, with most of the energy emitted lying in the NIR regions of the spectrum. Only 15–20% of the light falls into the visible range (400–700 nm), and less than 1% is ultraviolet light [24,25]. As a source of black-body radiation, a halogen bulb can be considered a rough approximation of a black-body radiator. A black-body radiator is a theoretical object that absorbs all the electromagnetic radiation that falls on it and emits radiation according to Planck’s law [26]. The main innovation lies in combining NIR and multispectral imaging with advanced image fusion techniques. In detail, Different wavelengths penetrate tissue with different paths to different depths, each providing specific information about tissue structure. By extracting valuable data from each image, the fused result offers enhanced detailed image.

## 2. Theoretical Explanation

Exposure fusion is a technique for synthesizing a single image from a series of input images by retaining only the regions corresponding to the highest-quality parameters defined in the model [17,27,28,29,30]. The process involves the computation of weight maps for each input image, where higher weights indicate that a pixel should contribute more prominently to the final composite image. These weights are designed to reflect desired image attributes, such as high contrast and optimal exposure. Unlike many other fusion techniques, exposure fusion does not depend on generating a high-dynamic-range (HDR) image [28]. This approach offers faster computational performance and is particularly advantageous for visualization on displays that do not support HDR format.

The quality parameters used for the weight calculations are as follows:

- Contrast: Contrast is typically measured using Michelson contrast [31] or RMS contrast [32]. However, in this application, a Laplacian filter was applied to the grayscale version of each image. The absolute value of the filter’s response was then calculated for each pixel. This method tends to assign higher weights to significant image features, such as edges and textures. This feature is denoted as *C* (the contrast weights) and is computed separately for each pixel in the image
- Exposure and Illumination power: Examining the unprocessed intensities within a channel allows us to assess the exposure quality of a pixel. Our objective is to retain intensities that are not close to zero (indicating underexposure) or one (indicating overexposure). We assign a weight to each intensity, denoted as *i*, based on its proximity to the pixel-normalized intensity middle value, 0.5, which is represented by employing a Gaussian curve:

(1) $$E=exp(-\frac{{\left(i-0.5\right)}^{2}}{{2\sigma }^{2}})$$

Here, *E* denotes the image exposedness per pixel, and *σ* (the standard deviation) controls the width of the Gaussian intensity distribution at each pixel. In our method, *σ* = 2.

Now we establish a weighted image parameter, *W*, in the *k*-th image defined as follows:

(2) $$W_{k}=C_{k}\cdot E_{k}$$

where *k* is the index of the image in a sequence. Furthermore, a weighted image, $\tilde{W}$, over the sequence of images is defined as:

(3) $$\tilde{W}=\sum_{n=1}^{N}W_{n}\;\;$$

where *N* is the number of images in the sequence. Then, the normalized weight per *k*-th image per each pixel is defined as follows:

(4) $$\hat{W_{k}}=\frac{W_{k}}{\tilde{W}}$$

The resulting fused image, *R*, can be defined as follows, calculated pixel-wise:

(5) $$\hat{R}=\sum_{n=1}^{N}\hat{W_{n}}\cdot I_{n}$$

where *I_n_* is the *n*-th image in the sequence.

**Figure 1:**
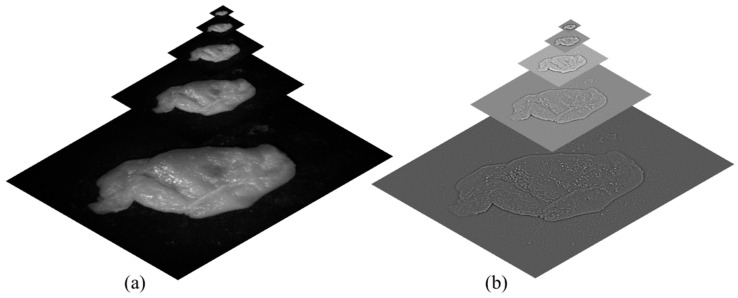
(**a**) Gaussian pyramid decomposition and (**b**) Laplacian pyramid decomposition.

Currently, the resulting image is of inadequate quality to properly depict the outcome. This issue arose because of the visible stitch lines, a consequence of rapid fluctuations in the weights. To overcome this issue, Laplacian pyramid decomposition was applied [33,34]. The Laplacian pyramid decomposition is a multi-scale image representation technique that decomposes an image into layers, each representing different levels of detail. It is built by iteratively applying a Gaussian filter to the image and down-sampling it by a factor of two, creating a sequence of images at progressively lower resolutions (known as the Gaussian pyramid). At each level, the higher-resolution image is reconstructed from the lower-resolution version using interpolation, and the difference (residuals) between the original and the reconstructed image is stored. These residuals form the layers of the Laplacian pyramid, which captures fine details at each scale. Figure 1 shows the concept of the Gaussian and Laplacian pyramid decomposition in the proposed research.

Following the Gaussian and Laplacian decompositions, there are *N* images along with *N* normalized weight maps functioning as alpha masks. The *l*-th level in a Laplacian pyramid decomposition of image *I* is represented as $L{\left\{I\right\}}^{l}$, while a Gaussian pyramid of image *I* is denoted as $G{\left\{I\right\}}^{l}$. The Laplacian pyramid of the image and the Gaussian pyramid of the normalized weights maps is pixel-wise multiplexed, as described in Equation (5):

(6) $$L{\left\{R\right\}}^{l}=\sum_{n=1}^{N}G{\left\{\hat{W}\right\}}_{n}^{l}L{\left\{I\right\}}_{n}^{l}$$

where *n* is the *n*-th image out of *N* images in the sequence. The computation of each level “*l*” within the resulting Laplacian pyramid involves a weighted average of the original Laplacian decompositions for that level. The weights are determined by the *l*-th level of the pyramid of the corresponding weight map. Finally, the pyramid, *L*{*R*}, collapses to derive *R*, which is the final fused image.

Our experiment involves two stages for capturing the initial sets of images: First, we capture ten images at a specific wavelength with varying exposures (ranging from underexposed to overexposed, evenly distributed). This procedure is subsequently repeated for ten distinct wavelengths. Consequently, in our experiment, *N* equals 100 (resulting from 10 wavelengths multiplied by 10 exposures each).

## 3. Experimental Setup

**Figure 2:**
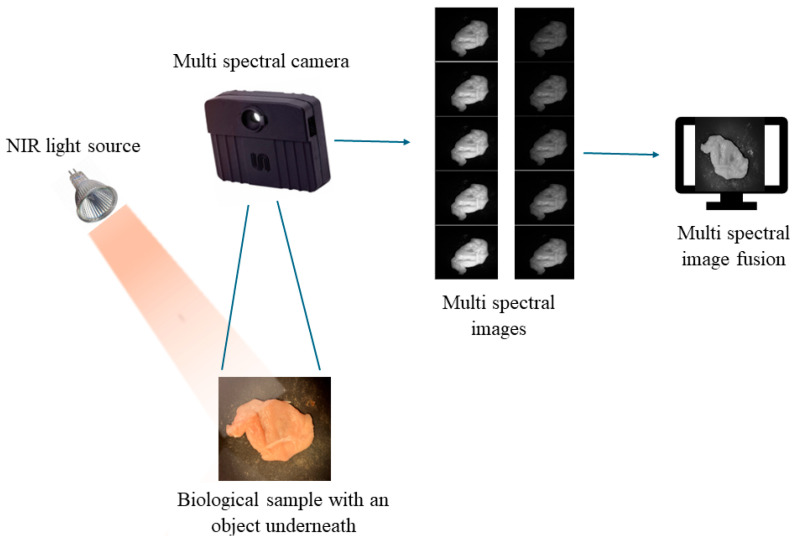
The schematic sketch of the setup.

The experimental setup was designed to capture images of back-reflection light coming from a sample situated under a halogen lamp using a hyperspectral camera with a wavelength range of 713 nm–920 nm (Monarch II hyperspectral, Unispectral, Ramat Gan, Israel, 1280 × 1024 Pixels, H-FOV (H, V, D) 31.5°, 25.5°, 39.8°, 10 CWL bands (±10 nm) 713, 736, 759, 782, 805, 828, 851, 874, 897, 920 nm). In Figure 2, one can see a schematic sketch of the setup. The camera was positioned perpendicular (90°) to the tissue with the light source angled at 70° towards an object that resembles the human body and with a denser material placed underneath (a piece of chicken breast with a plastic disk of 175 mm dia. underneath it) to simulate a tumor. The disk size in camera pixels was about 125 pixels in diameter. Both the object and denser material were placed on a non-reflective surface. The hyperspectral camera was positioned above the object (40 cm above the sample area), and images were taken in a dark environment. To ensure a uniform distribution of gray levels across the frame, we captured an image of a gray uniform patch before proceeding with the samples. The subsequent step involved acquiring the sequence of images. Then, a weight map was generated after considering the quality measures of each pixel in the images within the sequence. The final image was produced by consolidating the stack of images through weighted blending, with each image making a proportional contribution to the result determined by its weight in the weight map (Equations (1)–(6)).

## 4. Experimental Results and Discussion

**Figure 3:**
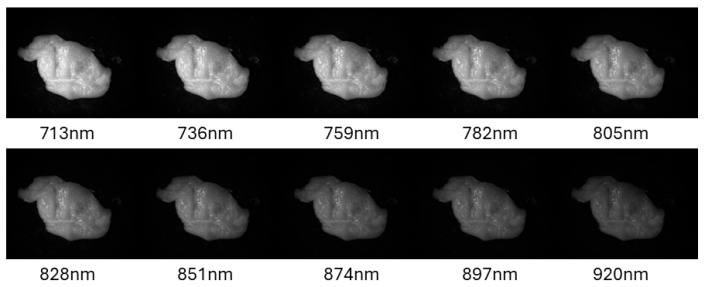
Experimental images: Hyperspectral images taken in a sequence with the same exposure of 100 ms. The corresponding filter wavelength of a specific image is shown below each one.

A standard CMOS camera, which records light wavelengths below the near-infrared (NIR) spectrum, would not be able to distinguish the plasticine underneath the chicken tissue. Consequently, a hyperspectral camera was used to acquire 10 sets of images at different wavelength bandwidths, with different 10 exposures per wavelength (100 images in total). The different exposures were taken to compensate for the uneven light intensities per wavelength of the light source. In Figure 3, one can see experimental images: hyperspectral images taken in a sequence. The object appears brighter at lower wavelengths because of detector sensitivity and the illumination spectrum. The corresponding filter wavelength of a specific image is mentioned below each one. The FWHM (Full-Width Half Maxima) of all images is 40 nm per bandwidth, with a spectral band accuracy of +/− 2 nm and a 100 ms exposure time. All the images presented in Figure 3 were taken at the same exposure time. One can see the different intensities due to the light source as mentioned above.

Subsequently, the image fusion technique was employed to merge all the images, aiming to generate a clearer and more harmonized composite. Following the merging of exposures, we iteratively applied the image fusion procedure to refine the detection of the plasticine area beneath the examined tissue, thereby facilitating a clearer delineation of its position. Furthermore, the illumination penetrating deeper into the region of interest (ROI) primarily comprises longer wavelengths within the near-infrared spectrum, undetectable by conventional silicon-based cameras.

To overcome this challenge, we utilized a camera equipped with near-infrared (NIR) detection capabilities. Leveraging this specialized camera, we captured reflections penetrating deeply into the tissue, thus revealing obscured objects. We acquired ten images from the hyperspectral camera for each exposure, spanning a wavelength range of 705–920 [nm], adequately suited for our objectives.

The purpose of the following step is to prepare all the images for the final stage of the fusion. After demonstrating that the camera can be used to detect objects behind scattering tissue, we wanted to improve its detection capability. In the first step, we captured the object at 10 different wavelengths as in the previous step (Figure 3). However, this time, we took sets of images with different exposures ranging from underexposed to overexposed (10 sets of images). The image fusion method was used to combine all the wavelengths into one image (as described before in Equations (1)–(6)). This method helped us extract the relevant information from each image and ignore any irrelevant information using the different interactions of each wavelength with the tissue. Since each wavelength reflection provides slightly different information about the area where the object is located, the combination of the images yielded a single image with more information, resulting in a clearer identification.

**Figure 4:**
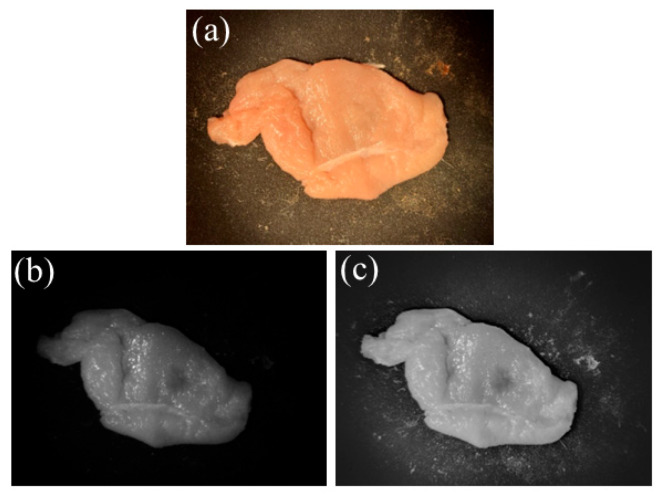
Experimental results: (**a**) white-light-illuminated image (RGB), (**b**) one-shot of NIR image, (**c**) resulting exposure fusion image.

In Figure 4, one can see the experimental results. In Figure 4a, a white-light-illumination image (RGB) is shown, and no signs of the internal phantom can be seen. In Figure 4b, a one-shot of the NIR image is shown, resolving some internal phantom image, and the resulting exposure fusion image is shown in Figure 4c, with a clear resolution of the internal tissue (a black spot in the middle of the image). Note that the gray-colored images are due to the monochromatic nature of the images.

**Figure 5:**
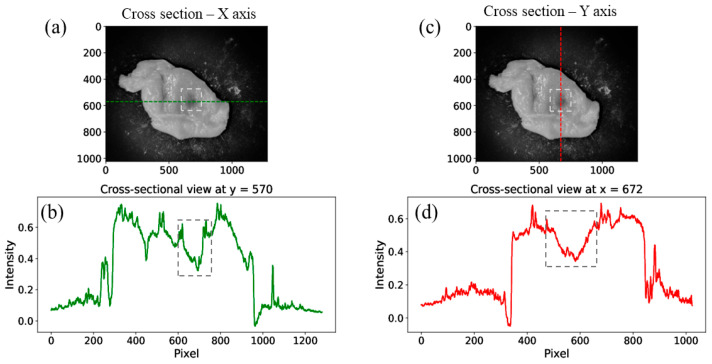
Images (**a**,**c**) are the fusion images, with green and red dashed lines indicating the cross-section’s locations. (**b**,**d**) Intensity profile along the X- and Y-axis at the black spot location along the dashed lines.

In Figure 5, one can see cross-sections of the fusion image that clearly show a black spot (tumor location) indicated on the phantom. Figure 5a,c show the fusion image with dashed lines indicating the X- and Y-axis cross-section locations, respectively. Figure 5b,d show the intensity profile along the X- and Y-axis at the black spot location (marked by the dashed lines). The green line denotes the horizontal axis intensity profile, while the red line denotes the vertical axis.

The resulting cross-sectional graphs presented enable us to clearly distinguish the presence of a high-density body beneath the diffusing tissue with a high level of certainty.

**Figure 6:**
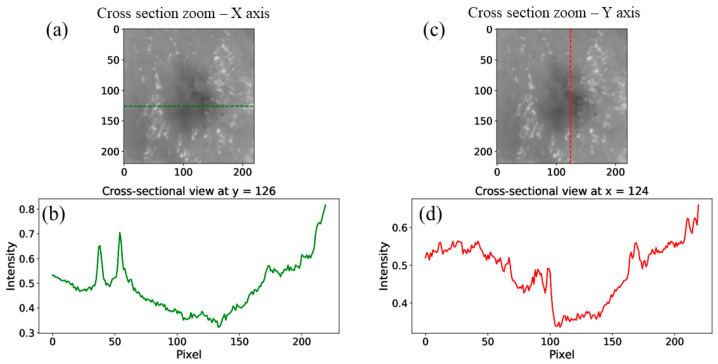
Experimental results: (**a**,**c**) zoomed version of the white dashed line areas in Figure 5 of the fusion image with the intensity profile along the Y-axis at the black spot location, (**b**,**d**) cross-section intensity profiles of the zoomed-in area.

In Figure 6, one can see a zoom-in of the fused image with the X- and Y-axis cross-section intensity profile (Figure 6a). The zoomed-in location is marked with dashed white lines, as can be seen in Figure 5.

The cross-sectional analysis presented in these images demonstrates the effectiveness of multispectral imaging and image fusion in detecting subsurface structures within diffusing biological tissues. The significant variations in intensity profiles along both the X- and Y-axis confirm the presence of a high-density object beneath the diffusing tissue.

Hyperspectral imaging with exposure fusion provides a powerful non-invasive method to visualize subsurface features in tissue. It is done by capturing a full reflectance spectrum at each pixel and combining multiple NIR wavelength images. It greatly enhances contrast and reveals hidden objects (e.g., tumors) that are indiscernible with conventional cameras. The proposed approach exploits the rich spectral and spatial information to differentiate materials by their optical signatures without any prior knowledge of the object, yielding superior delineation of the target in comparison with the background. However, the method is also characterized by high complexity and cost. It requires specialized NIR hyperspectral sensors, multi-exposure image acquisition and intensive data processing, resulting in large multi-dimensional datasets. Its performance is inherently limited to low tissue depths due to light attenuation, which collectively constrains its practical applicability. Our next step involves using deep convolutional neural networks to achieve the same results but using fewer images. Thus, we will need less computational and hyperspectral resources.

## 5. Conclusions

In this study, we demonstrated the feasibility of detecting reflections from objects situated within a dispersive layer using the near-infrared (NIR) range. Employing a hyperspectral camera operating within the NIR spectrum coupled with advanced image fusion techniques can enable the detection of cancerous tumors (phantoms) with improved resolution and clarity. By processing data from images captured across various bands of the near-IR range and merging them using image fusion methods, we devised a non-invasive and safe approach to tumor detection. This research lays the groundwork for future endeavors aimed at detecting cancerous tumors and blood vessels buried within tissue depths of the human body.

## Data Availability

Data is unavailable due to privacy or ethical restrictions.

## Abbreviations

The following abbreviations are used in this manuscript:

NIR	Near Infrared
MRI	Magnetic Resonance Imaging
CT	Computed Tomography
FWHM	Full-Width Half Maxima
RGB	Red, Green, Blue
